# The complete chloroplast genome sequence of *Thymus quinquecostatus* var. *japonicus* (Lamiaceae), an endemic to Ullenung Island of Korea

**DOI:** 10.1080/23802359.2020.1754942

**Published:** 2020-06-11

**Authors:** Hyoung Tae Kim, Jae-Hong Pak, Jung Sung Kim

**Affiliations:** a Institute of Agriculture Science and Technology, Chungbuk National University, Cheongju, Chungbuk, Republic of Korea; b Research Institute for Dokdo and Ulleungdo Island, Kyungpook National University, Daegu, Republic of Korea; c Department of Biology, Kyungpook National University, Daegu, Republic of Korea; d Department of Forest Science, Chungbuk National University, Cheongju, Chungbuk, Republic of Korea

**Keywords:** *Thymus quinquecostatus* var. *japonicus*, endemic species, Lamiaceae, chloroplast genome

## Abstract

The complete chloroplast (cp) genome sequence of *Thymus quinquecostatus* var. *japonicus,* an endemic species to Ulleung Island of Korea and used as plant material for folk remedies, was firstly analyzed in this study. It showed a typical circular structure composed of 151,782 bp in length and comprised of a large single-copy region (82,903bp) and a small single-copy region (17,667 bp) which were separated by two inverted repeat regions (25,606 bp). From the phylogenetic analyses of related taxa using the complete chloroplast genome sequences, it was proved that *T. quinquecostatus* var. *japonicus* is sister to the member of genus *Mentha* within the subfamily Nepetoideae.

The family Lamiaceae Martynov, well-known as mint family, is a widespread cosmopolitan plant group. Although the recognition and the classification of this larger family has been debated (Wagstaff and Olmstead 1997), it is recently regarded the plant group composed of 12 subfamilies and including over 7000 species (Chase et al. 2016). Among them, the genus *Thymus* L. belongs to the monophyletic subfamily Nepetoideae (Dumortier) Luerssonand including 250–350 taxa distributed mainly in Europe, Northwest Africa, and Asia (Lee et al. 2011; Bartolucci et al. 2013). For the commercial uses and medicinal feature, lots of study have been conducted to prove the components and effects using the extract from the plants (Lee et al. 2011). In Korea, three native species have been reported and *Thymus quinquecostatus* var. *japonicas* H. Hara is an plant endemic to Ulleung Island of Korea. The complete chloroplast genome of the vascular plants has been accumulated speedily, but there was no information about it of the genus *Thymus*. This is the first report of the complete chloroplast genome sequence of the genus.

We collected the plant material from Anpyungjeon (37°29.12′N, 130°52.5959′E, alt. 432.9 M) of Ulleung Island and the voucher (Lee & Kim 2017U4) was deposited at the Herbarium of Kyungpook National University (KNU). Complete chloroplast genome of *Thymus quinquecostatus* var. *japonicas* (MN867687)was sequenced by HiSeq4000 of Illumina. Totally 47,627,276 paired-end reads (2 × 151bp) were obtained and 39,328,684 reads were used for the assemble to the reference sequence after trimming with the length range 50–151 bp. The assembled reads were *de novo* assembled using the Geneious assembler. Using the assembled contigs, we aligned and repeated the procedure to make a single contig. Complete chloroplast genome was annotated using Geneious10.2.6 (Kearse et al., 2012) with manual correction and tRNAScan-SE (Lowe and Eddy 1997) for tRNA gene. The average coverage of this chloroplast genome was 859.7. The phylogenetic tree was constructed with related Lamiaceae members based on the concatenated 78 coding genes using RAxML (Stamatakis, 2014).

The complete chloroplast genome of *Thymus quinquecostatus* var. *japonicas* had a typical circular structure with 151,782 bp in length and comprised a large single-copy region (LSC, 82,903bp), a small single-copy region (SSC, 17,667bp), and two inverted repeat regions (IR, 25,606bp). The GC content was 37.9%. It was composed of 135 genes and 87 coding genes, 8 rRNA genes, and 37 tRNA genes were identified. In the phylogenetic tree conducted based on the protein-coding genes sequences, the monophyly of the subfamily Nepetoideae was strongly supported and the sister relationship between *Thymus quinquecostatus* var. *japonicus* and the genus *Mentha* was clearly proved (Figure 1). Both genera showed the close relationship to the genus *Dracocephalum* within the subfamily. Even though the phylogenetic relationship among the subfamilies of the family Lamiaceae still remains unclear, the generic relationship in the subfamily Nepetoideae and Lamioideae was suggested which have the amount of the data of complete chloroplast sequences.

**Figure 1.**
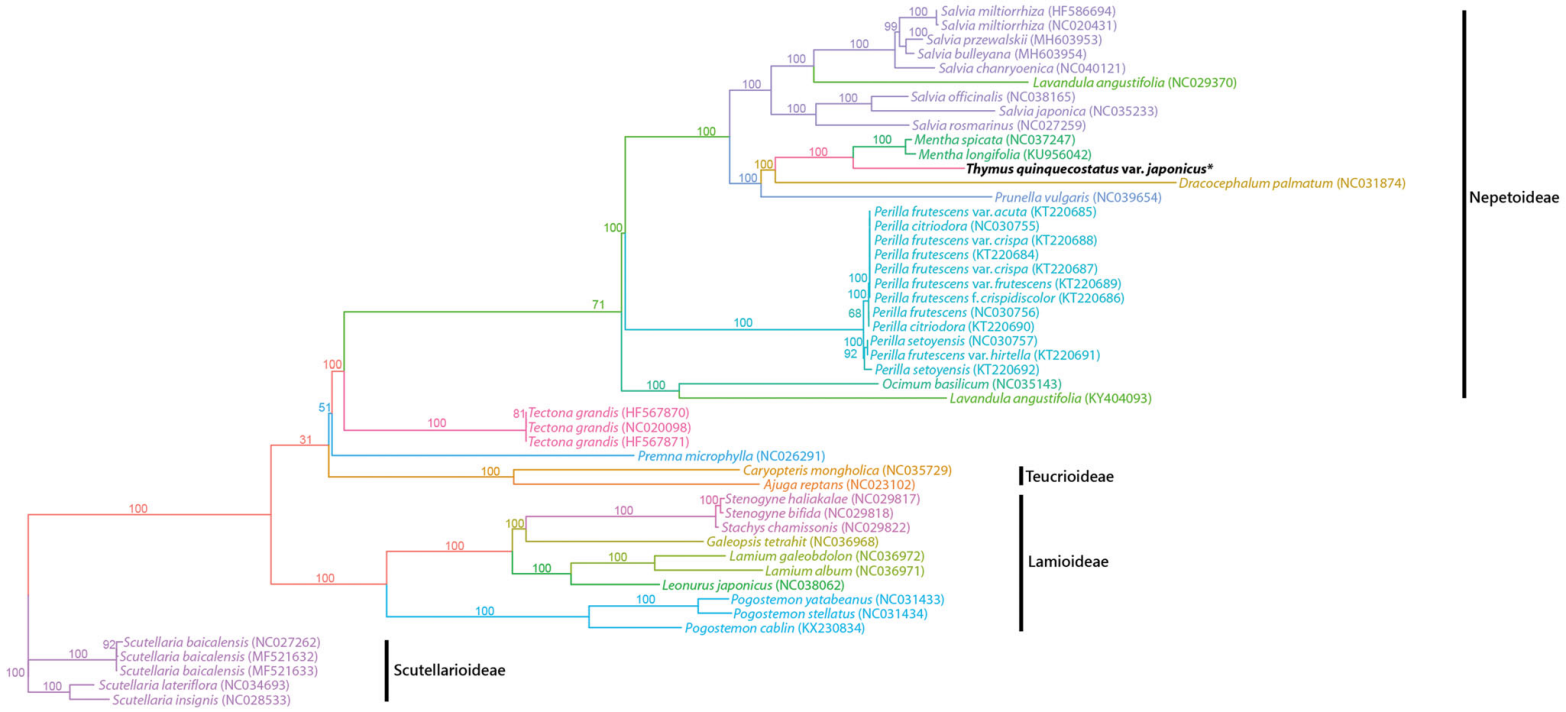
Phylogenetic tree of *Thymus quinquecostatus* var. *japonicus*and related taxa usingthe complete chloroplast genome sequences.

## Data Availability

The data that support the findings of this study are openly available in NCBI database at https://www.ncbi.nlm.nih.gov/, reference number [MN867687] after this article is published.
